# Obesity, Metabolic Syndrome and Risk of Atrial Fibrillation: A Swedish, Prospective Cohort Study

**DOI:** 10.1371/journal.pone.0127111

**Published:** 2015-05-15

**Authors:** Petter K. Nyström, Axel C. Carlsson, Karin Leander, Ulf de Faire, Mai-Lis Hellenius, Bruna Gigante

**Affiliations:** 1 Unit of Cardiovascular Epidemiology, Institute of Environmental Medicine, Karolinska Institutet, Stockholm, Sweden; 2 Centre for Family Medicine, Department of Neurobiology, Care Sciences and Society, Karolinska Institutet, Huddinge, Sweden; 3 Department of Medical Sciences, Molecular Epidemiology and Science for Life Laboratory, Uppsala University, Uppsala, Sweden; 4 Department of Cardiology, Karolinska University Hospital, Stockholm, Sweden; 5 Division of Cardiovascular Medicine, Department of Clinical Sciences, Danderyds Hospital, Karolinska Institutet, Stockholm, Sweden; Graduate School of Medicine, Osaka University, JAPAN

## Abstract

**Aim:**

We aimed to investigate whether different measures of obesity could similarly predict atrial fibrillation, and whether the atrial fibrillation risk associated with obesity is dependent on presence of metabolic syndrome.

**Material and Methods:**

We performed our study in a population-based longitudinal cardiovascular study, comprising 1 924 men and 2 097 women, aged 60 years, from Stockholm. Body mass index, waist circumference, sagittal abdominal diameter and components of metabolic syndrome (systolic- and diastolic blood pressure, fasting glucose, triglycerides, high-density lipoprotein-cholesterol) were recorded at baseline. Participants were classified by their body mass index (normal weight, overweight or obese), waist circumference (normal, semi-elevated or elevated), and according to presence of metabolic syndrome. Atrial fibrillation risk was estimated by Cox proportional hazards regression models, adjusted for common atrial fibrillation risk factors, expressed as HR and 95% CI.

**Results:**

During a mean follow-up of 13.6 years, 285 incident atrial fibrillation cases were recorded. One standard deviation increment of each obesity measure was associated with increased atrial fibrillation risk as: body mass index 1.25 (1.12 – 1.40), waist circumference 1.35 (1.19 – 1.54) and sagittal abdominal diameter 1.28 (1.14 – 1.44). Compared to normal weight subjects without metabolic syndrome, increased atrial fibrillation risk was noted for overweight subjects with metabolic syndrome, 1.67 (1.16 – 2.41), obese subjects without metabolic syndrome, 1.75 (1.11 – 2.74) and obese subjects with metabolic syndrome, 1.92 (1.34 – 2.74). Compared to subjects with normal waist circumference without metabolic syndrome, subjects with elevated waist circumference and metabolic syndrome suffered increased atrial fibrillation risk, 2.03 (1.44 – 2.87).

**Conclusions:**

Body mass index, waist circumference and sagittal abdominal diameter could similarly predict atrial fibrillation. Obesity was associated with an increased atrial fibrillation risk regardless of metabolic syndrome, whereas overweight and elevated waist circumference was associated with increased atrial fibrillation risk only if metabolic syndrome was present.

## Introduction

Obesity, a modifiable cardiovascular risk factor [1,2], has reached endemic proportions and has roughly doubled its world-wide prevalence since 1980 [3]. Obesity is also associated with an increased risk of atrial fibrillation (AF) [4], one of the most common atrial arrhythmias [5].

Several observational studies have shown that anthropometric measures of obesity body mass index (BMI) [6,7],waist circumference (WC) [6,8], hip circumference (HC) [6,8] and waist-hip ratio (WHR) [6] are incrementally associated with an increased risk of AF. However, it is unknown whether sagittal abdominal diameter (SAD), an alternate measure of abdominal obesity [9] strongly associated with CVD risk [10].

Risk of cardiovascular diseases (CVD) in obese individuals is partly dependent on the presence of the metabolic syndrome (MetS); a condition that, besides obesity, is characterized by impaired glucose homeostasis, high blood pressure, reduced high-density lipoprotein-cholesterol (HDL-C) and elevated triglycerides [11–13]. It was recently shown that the prevalence of MetS among obese adults ranges from 24% in Italian women to 78% in Finnish men [14]. MetS, defined as ≥ 3 of its components, is associated with an increased risk of atherosclerotic CVD [15,16], cardiovascular mortality [16] and AF [17–21]. In AF patients, MetS carries an additional risk of stroke beyond the risk predicted by the CHADS_2_ score [22]. Relevance of MetS as a CVD risk factor in obese individuals is supported by the observation that obese individuals without MetS, the so-called metabolically healthy obese (MHO) [14,23,24], are exposed to a risk of incident myocardial infarction (MI) that is not substantially higher than the risk of MI in normal weight individuals without MetS [25]. To the best of our knowledge, it is unknown to which extent the presence of MetS affects the risk of AF associated with obesity.

The aims of our study were to investigate whether different anthropometric measures of body size can equally predict AF and if the risk of AF associated with obesity is dependent on presence of MetS. The present study was performed in a large community-based prospective cardiovascular cohort, the 60-year-old men and women from Stockholm.

## Material and Methods

### Study population

From August 1997 to March 1999, every third man and woman living in Stockholm County, born between July 1^st^, 1937 and June 30^th^, 1938, was randomly selected from the population register in Sweden and invited to participate in a thorough health-screening study. Of the 5640 individuals (2681 women and 2779 men) invited to participate the response rate was 78%, resulting in 4232 participants (2181 women and 2033 men). Health screening included a physical examination, blood sampling after overnight fasting, a 12-lead ECG recording and an extensive questionnaire regarding lifestyle, health and indicators of socioeconomic status [26].

Incident cases of AF were obtained from the hospital discharge register in Sweden (100% follow-up), using codes of International Classification of Diseases, 10^th^ revision (ICD-10): I48.0, I48.1, I48.2, I48.3, I48.4 and I48.9, covering paroxysmal AF, persistent AF, chronic AF, typical atrial flutter type 1, atypical atrial flutter type 2, atrial- fibrillation and flutter unspecified. Atrial flutter was included, because the arrhythmia is closely related to AF and is associated with a similar risk of stroke [27]. Both main- and secondary diagnoses of AF were recorded. Extraction from the registers was done yearly until December 31^st^, 2012.

Participants were excluded from the present analysis if they had missing data regarding any measure of body size or MetS status (n = 135), were underweight (BMI < 18.5 kg/m^2^, n = 29), or had AF prior to enrollment (n = 47). Prior AF was defined as either AF on the baseline ECG, history of AF reported in the questionnaire or as ICD-10-code I48, obtained from the hospital discharge register in Sweden prior to enrollment date. The exclusion represented 5.0% of the original population, and 4021 participants remained eligible for analyses.

### Ethics

The cohort of 60 years old men and women from Stockholm was designed and conducted in accordance with the Helsinki Declaration. The study was approved 1996 (Dnr 96–398) by the Regional Ethics Review Board at Karolinska Institutet, Stockholm, Sweden. All the study participants gave their informed oral consent to be enrolled in the study, since at the time the study was initiated (1997) no forms for the written consent were available or in current use. Participation was voluntary and involved no evident risks and only study participants who agreed to participate in the study received the questionnaire and were invited for the medical examination. This consent procedure was approved by the Regional Ethics Review Board at Karolinska Institutet.

### Measures of body size and cardiometabolic parameters

Weight was obtained with an electronic scale, rounding the number to nearest 0.1 kg. Height was measured without shoes, rounding the number to nearest 0.5 cm. BMI was calculated as weight (kg), divided by the height (m) squared [28]. WC was measured after a normal expiration with patient standing up, wearing only underwear, rounding the number to nearest 0.5 cm. The measure was taken in the mid-point between the bottom of the lowest rib and the top of the iliac crest [9]. HC was measured at symphysis trochanter level [9] and the measure was rounded to nearest 0.5 cm. WHR was calculated by dividing WC in cm with HC in cm. SAD was measured after a normal expiration, with participant in supine position with straight legs and no clothes in the abdominal area. The measure was taken at the highest point of the belly, around the level of the iliac crest, with a ruler and a water level to ensure a vertical measure [29]. The measure was rounded to nearest 0.1 cm.

Systolic- and diastolic blood pressure were measured (HEM 711, OMROM Health Care) twice, in the right arm, after five minutes of rest, patient lying down. The mean of the two measures was calculated and rounded to nearest 0.1 mm Hg.

Venous blood samples were drawn after overnight fasting. Serum glucose was measured with an enzymatic colorimetric test (Bayer Diagnostics, Tarrytown, NY) and rounded to nearest 0.1 mmol/l, which was converted to mg/dl, by dividing values with 0.0555. Serum- cholesterol and triglycerides were analyzed using enzymatic methods (Bayer Diagnostics, Tarrytown, NY) and were rounded to nearest 0.1 mmol/l. Triglyceride levels were converted to mg/dl by dividing with 0.0113. Serum HDL-C was measured enzymatically, after isolation of low-density lipoprotein-cholesterol and very-low-density lipoprotein-cholesterol (Boehringer Mannheim GmbH, Germany), and rounded to nearest 0.01 mmol/l, which was converted to mg/dl by dividing values with 0.0259.

Medication for hypertension, diabetes and dyslipidemia was self-reported in the questionnaire. Birth country was self-reported and participants were classified as Swedish-born, or not Swedish-born. Smoking status was retrieved from the questionnaire and participants were classified as smokers or non-smokers. Consumption of alcoholic beverages was self-reported and converted to grams of alcohol per day [30]. Level of physical activity (PA) was self-reported and classified participants as performing regular exercise on at least moderate-intensity level, or not [31]. History of myocardial infarction (MI) was self-reported in the questionnaire, or recorded during follow-up, as codes of ICD-10: I21—I25, retrieved from the hospital discharge register in Sweden.

In the present study we have defined Mets according to the harmonized Mets definition reported in Alberti K et al [11]. In particular, this latest statement does not include obesity, defined by WC, as a mandatory criterion for MetS [12,13] and leaves the possibility to define obesity according to ethnicity and country specific guidelines. For the purpose of this analysis, MetS was defined as presence of ≥ 3 of the following: elevated WC [WC ≥ 102 cm in men and WC ≥ 88 cm in women], elevated triglycerides [triglycerides ≥ 150 mg/dl (1.7 mmol/l) or current medication for elevated triglycerides], reduced HDL-C [HDL-C < 40 mg/dl (1.9 mmol/l) in men, HDL-C < 50 mg/dl (1.3 mmol/l) in women, or current medication for reduced HDL-C], high blood pressure [systolic blood pressure (SBP) ≥ 130 mm Hg or diastolic blood pressure (DBP) ≥ 85 mm Hg or current use of antihypertensive medication] and elevated fasting glucose [fasting glucose ≥ 100 mg/dl (5.6 mmol/l) or current use of antidiabetic medication].

### Statistical analyses

Baseline characteristics of study participants were presented as means ± standard deviation (SD) for continuous variables and percentages for categorical variables. To determine differences between participants who developed AF and participants who did not, a Students t-test was used for continuous variables and a chi^2^-test was used for binary- and categorical variables. Baseline data was complete in all variables except birth-country (n = 3) smoking status (n = 60) and PA level (n = 57), in which a separate category “missing” was introduced.

Cox proportional hazards regression models were used to assess the risk of new-onset AF associated with obesity and MetS status. Time was defined as days from inclusion to either incident AF, or censoring through death or end of follow-up.

We analyzed the risk of new-onset AF associated with 1 standard deviation (SD) increment in weight, height, WC, HC, SAD, BMI and WHR. Risk was expressed as hazard ratios (HR) and 95% confidence intervals (CI). Crude risk estimates were adjusted by hypertension, elevated fasting glucose, sex, birth country (Sweden, not Sweden, missing), smoking status (smoker, non-smoker, missing), alcohol intake (g/day), regular moderate-intensity exercise (yes, no, missing) and history of MI.

To analyze the risk of AF associated with obesity and/or MetS we divided participants, according to their BMI and presence/absence of MetS, into 1 of 6 groups: normal weight (BMI 18.5–24.9 kg/m^2^) ± MetS, overweight (BMI 25.0–29.9 kg/m^2^) ± MetS and obese (BMI ≥ 30.0 kg/m^2^) ± MetS. HRs and 95% CIs were estimated, using normal weight subjects without MetS as reference. The multivariate model contained sex, birth country (Sweden, not Sweden, missing), smoking status (smoker, non-smoker, missing), alcohol intake (g/day), regular moderate-intensity exercise (yes, no, missing) and history of MI.

WC is the measure of obesity used in the MetS definition. Therefore, we also analyzed the risk of AF based upon WC level. In this analysis, elevated WC was excluded as a MetS component, and presence of ≥ 2 of the remaining 4 MetS components was regarded as Mets. Participants were classified according to their WC and presence/absence of MetS, into 1 of 6 groups: normal WC (< 94.0 cm in men, < 80.0 cm in women) ± MetS, semi-elevated WC (94.0–101.9 cm in men, 80.0–87.9 cm in women) ± MetS and elevated WC (≥ 102.0 cm in men, ≥ 88.0 cm in women) ± MetS. The WC interval defined as semi-elevated WC represents a WC level associated with increased risk for metabolic complications in Caucasian populations [32]. HRs and 95% CIs were estimated, using subjects with normal WC without MetS as reference. The multivariate model contained sex, birth country (Sweden, not Sweden, missing), smoking status (smoker, non-smoker, missing), alcohol intake (g/day), regular moderate-intensity exercise (yes, no, missing) and history of MI.

Finally, in a secondary analysis, we assessed the risk of new-onset AF according to presence of each MetS component within each BMI- and WC-interval. HRs and 95% CIs were estimated using participants from the same BMI/WC-group without the actual MetS component, as the reference. The multivariate model contained sex, birth country (Sweden, not Sweden, missing), smoking status (smoker, non-smoker, missing), alcohol intake (g/day), regular moderate-intensity exercise (yes, no, missing), history of MI and the other components of MetS.

Significance level was set at a p-value < 0.05. All statistical analyses were performed with STATA 11 software (Stata Corporation, College Station, TX, USA).

## Results

A total of 4021 participants, of which 1924 (47.9%) were men, were eligible for analyses. MetS was present in 1 108 participants (27.6%) and 795 participants (19.8%) were classified as obese according to their BMI. Of the 4021 participants eligible for analyses, 3454 were alive at end of-follow-up and the mortality rate was 14.1%. Among those who died, CVD was the cause of death in 27.9% of cases. The proportion of study participants with MetS at baseline who were alive at the end of the follow up was 27.6%, as compared to 25.3% among those who died from CVD and 27.9% among those who died from other causes.

During follow-up, which on average was 13.6 years, 285 cases of incident AF were registered and the incidence rate was 5.2 per 1 000 person-years. Characteristics of the participants who developed AF and those who did not are summarized in Table 1 and in S1 Table. Participants who developed AF were more often men, had a higher prevalence of MetS, higher alcohol intake and were “bigger”, regardless of anthropometric measure used to assess body size. In both groups, hypertension was the most prevalent MetS component and elevated triglycerides was the least common.

**Table 1:** Baseline characteristics of study population.

	**No atrial fibrillation (n = 3736)**	**Incident atrial fibrillation (n = 285)**	**P**
Male sex, %	47.2	56.1	0.004
Weight, kg	77.2 ± 13.9	83.7 ± 15.8	< 0.001
Height, cm	169.6 ± 9.0	173.0 ± 9.1	< 0.001
Body mass index, kg/m^2^	26.8 ± 4.1	28.0 ± 4.9	< 0.001
Hip circumference, cm	103.3 ± 8.2	106.4 ± 9.6	< 0.001
Waist-hip ratio	0.89 ± 0.09	0.90 ± 0.09	< 0.002
Sagittal abdominal diameter, cm	20.6 ± 2.8	21.5 ± 3.3	< 0.001
Waist circumference men, cm	97.5 ± 10.2	100.7 ± 11.7	< 0.001
Waist circumference women, cm	86.5 ± 11.6	90.5 ± 14.3	< 0.001
Elevated waist circumference, %	36.8	45.6	0.003
Hypertension, %	61.0	75.4	< 0.001
Elevated fasting glucose, %	30.4	34.4	0.160
Reduced HDL-cholesterol, %	23.0	28.4	0.038
Elevated triglycerides, %	22.4	26.7	0.100
Components of metabolic syndrome	1.7 ± 1.3	2.1 ± 1.4	< 0.001
Metabolic syndrome ^a^, %	26.8	37.9	< 0.001
Swedish-born, %	81.4	79.0	0.514
Current smoker, %	21.0	22.8	0.212
Alcohol intake, g/day	12.8 ± 14.2	15.2 ± 16.3	0.007
Physically active, %	30.1	30.5	0.580
Prior myocardial infarction, %	5.3	5.6	0.804

Continuous variables presented as means ± standard deviations.

^a^ Presence of ≥ 3 of: elevated waist circumference, hypertension, elevated fasting glucose, elevated triglycerides, reduced HDL-C.

Table 2 illustrates the risk estimates of incident AF, associated with increase of 1 SD in anthropometric measures of body size. Weight, height, WC, HC, SAD, BMI and WHR were positively associated with the risk of AF in univariate models, but the risk associated with WHR did not remain significant in the multivariate model. The highest risk estimate was observed for height and the risk estimates of the other measures were in the same order of magnitude.

**Table 2:** AF risk associated with 1 SD increment in anthropometric measures of body size.

Measure of body size	Univariate modelHR (95% CI) ^a^	P	Multivariate model ^b^HR (95% CI) ^a^	P
Weight	1.50 (1.35–1.66)	< 0.001	1.47 (1.31–1.65)	< 0.001
Height	1.45 (1.29–1.63)	< 0.001	1.72 (1.44–2.04)	< 0.001
WC	1.43 (1.28–1.59)	< 0.001	1.35 (1.19–1.54)	< 0.001
HC	1.34 (1.22–1.48)	< 0.001	1.38 (1.24–1.53)	< 0.001
SAD	1.37 (1.23–1.52)	< 0.001	1.28 (1.14–1.44)	< 0.001
BMI	1.28 (1.15–1.42)	< 0.001	1.25 (1.12–1.40)	< 0.001
WHR	1.25 (1.11–1.41)	< 0.001	1.05 (0.88–1.25)	0.588

Abbreviations: BMI, body mass index; CI, confidence interval; HC, hip circumference; HDL-C, high-density lipoprotein-cholesterol; HR, hazard ratio; MI, myocardial infarction; SAD, sagittal abdominal diameter; WC, waist circumference; WHR, waist-hip ratio.

^a^ Expressed per 1 SD increment of each measure: Weight = 14.2 kg, Height = 9.1 cm, WC = 12.4 cm, HC = 8.4 cm, SAD = 2.9 cm, BMI = 4.2 kg/m^2^, WHR = 0.087.

^b^ Model containing: hypertension, elevated fasting glucose, sex, smoking (yes, no, missing), alcohol intake (g/day), history of MI, regular moderate-intensity PA (yes, no, missing), and Swedish-born (yes, no, missing).

The risk estimates of incident AF associated with BMI classification and presence of MetS, as compared to the normal weight participants without MetS, are reported in Table 3. Increased risk of AF was noted for overweight individuals with MetS, in uni- and multivariate models. Obesity, instead, was associated with an increased risk of AF regardless of MetS and obese subjects with MetS suffered only a marginally higher risk of AF than obese subjects without MetS.

**Table 3:** AF risk according to BMI categories and presence of MetS.

	Univariate model HR (95% CI)	P	Multivariate model ^c^ HR (95% CI)	P
Normal weight ^a^, no MetS (n = 1 334)	Reference	-	Reference	-
Normal weight, MetS ^b^ (n = 92)	1.22 (0.53–2.81)	0.637	1.17 (0.51–2.70)	0.713
Overweight ^a^, no MetS (n = 1 295)	1.04 (0.76–1.44)	0.790	1.01 (0.73–1.40)	0.949
Overweight, MetS ^b^ (n = 505)	1.81 (1.26–2.60)	0.001	1.67 (1.16–2.41)	0.006
Obese ^a^, no MetS (n = 284)	1.67 (1.07–2.62)	0.024	1.75 (1.11–2.74)	0.015
Obese, MetS ^b^ (n = 511)	2.01 (1.41–2.85)	< 0.001	1.92 (1.34–2.74)	< 0.001

Abbreviations: BMI, body mass index; CI, confidence interval; HDL-C, high-density lipoprotein-cholesterol; HR, hazard ratio; MetS, metabolic syndrome; MI; myocardial infarction; PA, physical activity; WC, waist circumference.

^a^ Normal weight, BMI 18.5–24.9 kg/m^2^; overweight BMI 25.0–29.9 kg/m^2^; obese BMI ≥ 30.0 kg/m^2^.

^b^ Presence of ≥ 3 of: elevated WC, hypertension, elevated fasting glucose, elevated triglycerides, reduced HDL-C.

^c^ Model containing: sex, birth-country (Sweden, not Sweden, missing), smoking status (yes, no, missing), alcohol intake (g/day), regular moderate-intensity PA (yes, no, missing) and history of MI.

Table 4 shows the risk estimates of incident AF associated with degree of obesity, defined by WC level, and the presence or absence of MetS. Subjects with normal WC and no MetS were used as referent group. Increased risk of AF was observed only in subjects with elevated WC and MetS.

**Table 4:** AF risk associated with increasing WC and presence of MetS.

	Univariate model HR (95% CI)	P	Multivariate model ^c^ HR (95% CI)	P
Normal WC ^a^, no MetS (n = 1041)	Reference	-	Reference	-
Normal WC, MetS ^b^ (n = 281)	1.59 (0.97–2.61)	0.067	1.45 (0.88–2.38)	0.148
Semi-elevated WC ^a^, no MetS (n = 723)	1.17 (0.79–1.75)	0.431	1.19 (0.80–1.78)	0.385
Semi-elevated WC, MetS ^b^ (n = 473)	1.48 (0.97–2.27)	0.070	1.33 (0.86–2.04)	0.196
Elevated WC ^a^, no MetS (n = 634)	1.30 (0.87–1.94)	0.207	1.37 (0.91–2.07)	0.129
Elevated WC, MetS ^b^ (n = 869)	2.09 (1.49–2.93)	< 0.001	2.03 (1.44–2.87)	< 0.001

Abbreviations: CI, confidence interval; HDL-C, high-density lipoprotein-cholesterol; HR, hazard ratio; MetS, metabolic syndrome; MI; myocardial infarction; PA, physical activity; WC, waist circumference.

^a^ Normal WC: < 94.0 cm in men, < 80.0 cm in women; Semi-elevated WC: 94.0–101.9 cm in men, 80.0–87.9 cm in women. Elevated WC: ≥ 102.0 cm in men, ≥ 88.0 cm in women.

^b^ Presence of ≥ 2 of: hypertension, elevates fasting glucose, elevated triglycerides, reduced HDL-C.

^c^ Model containing: sex, birth-country (Sweden, not Sweden, missing), smoking status (yes, no, missing), alcohol consumption (g/day), regular moderate-intensity PA (yes, no, missing) and history of MI.

Finally, we have analyzed the risk of incident AF, associated with presence of each one of the MetS components within each BMI interval (Table 5) as well as within each WC interval (Table 6). As summarized in Table 5, only hypertension was associated with an increased risk of AF in normal weight- and overweight participants, but did not significantly modify AF risk in the obese individuals. The other MetS components alone were not associated with the risk of AF in any BMI interval.

**Table 5:** AF risk estimates by BMI category and presence of each one of the components of MetS.

	Univariate model HR (95% CI)	P	Multivariate model ^b^ HR (95% CI)	P
Normal weight ^a^, elevated WC (3.2) ^c^	0.78 (0.19–3.18)	0.731	0.88 (0.21–3.70)	0.865
Normal weight, hypertension (49.6)	2.12 (1.33–3.37)	0.001	2.10 (1.31–3.37)	0.002
Normal weight, elevated fasting glucose (19.4)	1.19 (0.69–2.03)	0.534	1.05 (0.61–1.83)	0.858
Normal weight, elevated triglycerides (10.7)	0.95 (0.46–1.97)	0.891	0.87(0.40–1.87)	0.714
Normal weight, reduced HDL-C (13.4)	0.95 (0.49–1.85)	0.889	1.00 (0.50–2.01)	0.997
Overweight ^a^, elevated WC (39.1)	1.11 (0.78–1.58)	0.569	1.14 (0.78–1.67)	0.501
Overweight, hypertension (66.3)	1.93 (1.26–2.95)	0.003	1.69 (1.09–2.62)	0.020
Overweight, elevated fasting glucose (31.4)	1.24 (0.86–1.79)	0.253	0.99 (0.68–1.45)	0.975
Overweight, elevated triglycerides (24.6)	1.44 (0.98–2.11)	0.061	1.17 (0.77–1.76)	0.460
Overweight, reduced HDL-C (24.8)	1.33 (0.91–1.96)	0.144	1.23 (0.81–1.85)	0.335
Obese ^b^, elevated WC (98.8)	1.47 (0.46–4.66)	0.512	1.39 (0.43–4.52)	0.585
Obese, hypertension (74.7)	1.45 (0.84–2.51)	0.185	1.33 (0.75–2.36)	0.332
Obese, elevated fasting glucose (49.3)	0.87 (0.56–1.35)	0.525	0.77 (0.49–1.21)	0.262
Obese, elevated triglycerides (40.3)	0.91 (0.58–1.43)	0.681	0.81 (0.50–1.30)	0.377
Obese, reduced HDL-C (38.2)	1.22 (0.78–1.90)	0.384	1.34 (0.83–2.15)	0.226

Abbreviations: BMI, body mass index; CI, confidence interval; HDL-C, high-density lipoprotein-cholesterol; HR, hazard ratio; MetS, metabolic syndrome; MI; myocardial infarction; PA, physical activity; WC, waist circumference.

^a^ Normal weight, BMI 18.5–24.9 kg/m^2^; overweight BMI 25.0–29.9 kg/m^2^; obese BMI ≥ 30.0 kg/m^2^.

^b^ Model containing: sex, birth-country (Sweden, not Sweden, missing), smoking status (yes, no, missing), alcohol consumption (g/day), regular moderate-intensity PA (yes, no, missing), history of MI and the other components of MetS.

^**c**^ Percentage of the participants with the MetS component, within the given BMI interval.

**Table 6:** AF risk estimates by increasing WC and presence of each one of the components of MetS.

	Univariate model HR (95% CI)	P	Multivariate model ^b^ HR (95% CI)	P
Normal WC ^a^, hypertension (50.3) ^c^	2.61 (1.59–4.29)	< 0.001	2.54 (1.54–4.21)	< 0.001
Normal WC, elevated fasting glucose (19.4)	0.97 (0.54–1.73)	0.907	0.75 (0.42–1.36)	0.351
Normal WC, elevated triglycerides (9.3)	1.01 (0.46–2.19)	0.984	0.80 (0.35–1.83)	0.594
Normal WC, reduced HDL-C (11.7)	1.02 (0.51–2.05)	0.948	1.31 (0.54–2.36)	0.741
Semi-elevated WC ^a^, hypertension (61.9)	2.14 (1.27–3.62)	0.004	1.92 (1.10–3.33)	0.021
Semi-elevated WC, elevated fasting glucose (27.6)	1.03 (0.63–1.68)	0.908	0.87 (0.52–1.45)	0.596
Semi-elevated WC, elevated triglycerides (22.2)	1.25 (0.75–2.08)	0.388	1.12 (0.66–1.92)	0.677
Semi-elevated WC, reduced HDL-C (24.0)	1.04 (0.62–1.75)	0.869	0.93 (0.54–1.59)	0.780
Elevated WC ^a^, hypertension (72.5)	1.33 (0.89–2.00)	0.168	1.18 (0.77–1.79)	0.449
Elevated WC, elevated fasting glucose (43.1)	1.25 (0.88–1.76)	0.208	1.07 (0.75–1.53)	0.720
Elevated WC, elevated triglycerides (35.0)	1.18 (0.83–1.68)	0.367	0.95 (0.65–1.39)	0.800
Elevated WC, reduced HDL-C (33.2)	1.44 (1.01–2.04)	0.042	1.39 (0.95–2.03)	0.087

Abbreviations: BMI, body mass index; CI, confidence interval; HDL-C, high-density lipoprotein-cholesterol; HR, hazard ratio; MetS, metabolic syndrome; MI; myocardial infarction; PA, physical activity; WC, waist circumference.

^a^ Normal WC: < 94.0 cm in men, < 80.0 cm in women; Semi-elevated WC: 94.0–101.9 cm in men, 80.0–87.9 cm in women. Elevated WC: ≥ 102.0 cm in men, ≥ 88.0 cm in women.

^b^ Model containing: sex, birth-country (Sweden, not Sweden, missing), smoking status (yes, no, missing), alcohol consumption (g/day), regular moderate-intensity PA (yes, no, missing), history of MI and the other components of MetS.

^c^ Percentage of the participants with the MetS component, within the given WC interval.

Similarly, when the analysis was performed using WC to define obesity (Table 6), hypertension was the only MetS component associated with an increased risk of AF, in participants with normal or semi-elevated WC. Reduced HDL-C was associated with an increased risk of AF in participants with elevated WC in the univariate model, but not in the multivariate model.

## Discussion

Our results show that anthropometric measures of obesity, except WHR, can similarly predict AF risk independently from other known AF risk factors and that obesity defined as a BMI ≥ 30.0 is associated with an increased risk of AF regardless of MetS. In individuals with overweight, defined as a BMI 25.0–29.9, or abdominal obesity, defined as an elevated WC, increased risk of AF was observed only if MetS was present.

### Anthropometric measures of body size and risk of atrial fibrillation

Our results mainly support previous findings showing an association of increases in weight [6,33,34], height [6,33,34], WC [6,8], HC [6,8], BMI [4,6,7] and WHR [6], with the exception from WHR, which was not associated with the risk of AF in multivariate models. Aside from WHR, the anthropometric measures of obesity could equally predict AF risk, independently from other known cardiovascular risk factors. Since the denominator of WHR, HC, was itself strongly associated with an increased risk of AF, it was however expected that WHR could not predict AF risk. The other anthropometric measures of obesity investigated in the present study could equally predict AF risk, independently from other known cardiovascular risk factors.

Weight, height, body fat mass [35–37] and BMI [7,38] are all incrementally associated with left atrial volume. Left atrial enlargement is in turn, regardless of left ventricular size, an independent precursor of AF through founding and prolongation of ectopic signals from the atrial tissue [36,37]. Thus, left atrium enlargement is a probable effect mediator between increased body size, regardless of how it is measured, and the development of AF.

In the present study we have also, for the first time, investigated SAD as a predictor of AF and found that SAD is incrementally associated with an increased risk of AF. WC and SAD are both measures of abdominal obesity, but SAD correlates better with MetS [39], CVD risk factors [40], incident CVDs [10] and the amount of visceral- [41] and epicardial [42] adipose tissue. Since visceral- and epicardial adipose tissue have a high secretion of inflammatory mediators, adipokines [40], and because inflammation is associated with the risk of AF [43], we hypothesized that SAD would have a higher predictive value of AF risk than WC. However, the association with the AF risk was similar in strength between the two measures with a small advantage, if any, of WC in favor of SAD. However, the association of obesity with risk of AF is complex and probably not directly related to the body distribution of adipose tissue. In fact, recent data from a Danish cohort indicates that not only fat mass, but even fat free mass, derived from bioelectrical impedance, was associated with an increased risk of AF [6]. Although fat mass and fat free mass seem to correlate [6], more sensitive imaging modalities might be necessary [40] to fully understand how different types of tissue mass affect the risk of AF.

### Obesity, metabolic syndrome and risk of atrial fibrillation

In the present study, we found that obesity, defined as a BMI ≥ 30.0, was associated with an increased risk of AF regardless of MetS. Nevertheless, overweight, defined as a BMI 25.0–29.9 was associated with an increased risk of AF only if MetS was present. These results were consistent regardless of whether elevated WC was included as a component of MetS or not. In a previous meta-analysis of 5 population-based cohorts with 78 602 subjects [4], the relative risk of AF was 1.39 for overweight- and 1.87 for obese subjects, as compared to normal weight subjects, defined as a BMI 18.5–24.9. We found risk estimates of similar magnitude in obese subjects, but we further suggest that sole overweight is not independently associated with AF risk, but dependent on MetS.

In contrast to obesity defined by BMI, we found that abdominal obesity defined as an elevated WC, was not associated with the risk of AF unless other commonly accompanying MetS components were present. Neither was MetS associated with the risk of AF in subjects without an elevated WC. Previous observational studies have described that an elevated WC [18,19], as well as MetS [17–19], is associated with the risk of AF.

Although BMI and WC are both obesity measures, closely correlating in the present cohort, the cutoff levels to define obesity targeted different groups of participants. Nearly all subjects with BMI ≥ 30.0 had also an elevated WC, but only half of the subjects with an elevated WC had also a BMI ≥ 30.0. Thus, the two groups of obese individuals, defined by the two measures, were not equivalent. In particular, the group of subjects with an elevated WC contained a high proportion of overweight subjects (BMI 25.0–29.9) and this presumably explains why this group of individuals was at increased risk of AF only when MetS was present.

When analyzing individual components of MetS, hypertension was the only individual MetS component associated with the risk of AF, in subjects defined as non-obese by either BMI or WC. Meanwhile hypertension is an established risk factor for AF [44–46] it has previously been questioned whether elevated triglycerides [17–20], reduced HDL-C [47] and type 2 diabetes mellitus [48,49] have an impact on the AF risk in the general population. Consistently with previous findings, our results suggest that the association between MetS and the risk of AF in non-obese subjects is primarily dependent on presence of hypertension, while hypertension seems not to increase the AF risk associated with obesity.

### Strengths and limitations

Our study was a population-based cohort, with a high participation rate, an equal number of men and women and an almost complete follow-up (99.7%) from Swedish registers [50]. The study participants were asked to fill in a comprehensive questionnaire and participated in a detailed medical examination which allowed us to thoroughly characterize the participants and adjust for potential confounders. Of note, all participants were of the same age, thus eliminating potential age as potential confounding factor.

AF diagnosis was ascertained only via the hospital discharge register while data from Swedish primary health care centers were not available for the present analysis. A corollary of this limitation is that the risk of AF in obese individuals could have been overestimated if obese individuals had a higher hospitalization rate. However, the proportion of AF reported as a secondary diagnosis did not differ between subjects with a BMI ≥ 30.0 (0.45) as compared to subjects with a BMI < 30.0 (0.46), and the risk estimates we report are in line with those reported in a recently published meta-analysis [4]. Also, it was recently described that of all AF diagnoses recorded in Stockholm County between years 2006 and 2010, only 12% were recorded in primary care exclusively [51]. Both AF and atrial flutter were used as main outcomes in the present analysis. While this might be correct from a clinical point of view since these two arrhythmias often coexist in the same patient and are associated with a similar risk of thromboembolic events [27], we cannot exclude that they might differ to a certain extent from an etiological point of view. Larger studies with a more detailed classification of the arrhythmia in the single patient are warranted to better define similarities and differences between these two clinical entities.

Our cohort had a high number of Swedish-born participants (81.2%) and extrapolation of our findings to other ethnic groups should be done with caution. Although we adjusted our results for several known confounders, we cannot exclude the presence of residual confounding as well as unmeasured factors. In particular, we lacked data on left atrium size, the inflammation marker C-reactive protein (CRP), body fat and cardiorespiratory fitness (CRF). Left atrium enlargement and systemic inflammation are both probable effect mediators between obesity and the development of AF and low levels of CRP has been suggested to play a role in the protective profile of MHO individuals [23]. Body fat is associated with the AF risk [6], but also with a better prognosis in subjects with coronary heart disease [52] and heart failure [53]. CRF is also associated with a better prognosis in patients with CVD [52,54] or heart failure [52]. Also, a recent meta-analysis indicates that a good CRF level is associated with a decreased risk of all-cause mortality in a general population, regardless of BMI [55]. Data on body fat mass and CRF would have allowed us a better definition of the obese phenotype associated with an increased risk of AF.

## Conclusions

In the present study of 60 years old men and women we found that weight, height, WC, HC, SAD and BMI were similarly, incrementally, associated with an increased risk of AF. In order to screen for AF risk, any of these measures should suffice. Based on our results we would however not recommend WHR [10], to estimate AF risk.

We also found that subjects with general obesity (BMI ≥ 30.0) were at increased risk of AF regardless of MetS, meanwhile subjects with overweight (BMI 25.0–29.9) or abdominal obesity, defined as an elevated WC, (WC ≥ 102 cm in men, ≥88 cm in women) suffered an increased AF risk only if MetS was present.

Further studies are required to confirm our findings in non-Scandinavian populations as well as to evaluate the relevance of inflammatory- and metabolic biomarkers as risk predictors of AF in non-obese individuals.

## Supporting Information

S1 Table Additional baseline characteristics of the study participants. (DOCX)
