# Supplementary material for: Obesity, Metabolic Syndrome and Risk of Atrial Fibrillation: A Swedish, Prospective Cohort Study
Source: PLoS One. 2015 May 15;10(5):e0127111. doi: 10.1371/journal.pone.0127111 (PMC4433194; doi:10.1371/journal.pone.0127111)
Supplement: S1 Table — (DOCX) [file pone.0127111.s001.docx]

# S1 Table. Additional baseline characteristics of the study participants

|  | No atrial fibrillation  (n = 3 736) | Incident atrial fibrillation (n = 285) | P |
| --- | --- | --- | --- |
| BMI group (normal weight, overweight, obese), % | 36.0, 44.8, 19.2 | 28.1, 43.8, 28.1 | < 0.001 |
| WC interval (normal, semi-elevated, elevated), % | 33.3, 29.9, 36.8 | 26.7, 27.7, 45.6 | 0.008 |
| Systolic blood pressure, mm Hg | 137.9 ± 21.7 | 143.8 ± 21.9 | < 0.001 |
| Diastolic blood pressure, mm Hg | 84.3 ±10.6 | 87.0 ± 10.7 | < 0.001 |
| Antihypertensive medication, % | 18.0 | 34.4 | < 0.001 |
| Fasting glucose, mmol/l | 5.53 ± 1.61 | 5.71 ± 1.82 | 0.074 |
| Fasting glucose, mg/dl | 99.58 ±29.01 | 102.80 ± 32.85 | 0.074 |
| Antidiabetic medication, % | 3.2 | 6.0 | 0.014 |
| LDL-C, mmol/l | 3.88 ± 0.92 | 3.80 ± 1.00 | 0.173 |
| Apolipoprotein A1, mmol/l | 1.53 ± 0.27 | 1.53 ± 0.26 | 0.962 |
| Apolipoprotein B, mmol/l | 1.07 ± 0.23 | 1.06 ± 0.24 | 0.770 |
| Lipid-altering medication, % | 4.8 | 10.2 | < 0.001 |
| Fibrinogen, mmol/l | 3.03 ± 0.77 | 3.11 ± 0.82 | 0.089 |
| Employment status (employed, early retired, unemployed), % | 76.6, 15.8, 7.6 | 76.6, 16.5, 6.9 | 0.891 |

Abbreviations: BMI, body mass index; LDL-C, low-density lipoprotein-cholesterol; WC, waist circumference.

Continuous variables presented as means ± standard deviations.
